# A Human Blood-Brain Barrier Transcytosis Assay Reveals Antibody Transcytosis Influenced by pH-Dependent Receptor Binding

**DOI:** 10.1371/journal.pone.0096340

**Published:** 2014-04-30

**Authors:** Hadassah Sade, Claudia Baumgartner, Adrian Hugenmatter, Ekkehard Moessner, Per-Ola Freskgård, Jens Niewoehner

**Affiliations:** 1 Large Molecule Research, Pharma Research and Early Development (pRED), Roche, Penzberg, Germany; 2 Large Molecule Research, Pharma Research and Early Development (pRED), Roche, Schlieren, Switzerland; 3 Neuroscience Discovery and Translation Area, Pharma Research and Early Development (pRED), F. Hoffmann-La Roche, Basel, Switzerland; University of Regensburg, Germany

## Abstract

We have adapted an *in vitro* model of the human blood-brain barrier, the immortalized human cerebral microvascular endothelial cells (hCMEC/D3), to quantitatively measure protein transcytosis. After validating the receptor-mediated transport using transferrin, the system was used to measure transcytosis rates of antibodies directed against potential brain shuttle receptors. While an antibody to the insulin-like growth factor 1 receptor (IGF1R) was exclusively recycled to the apical compartment, the fate of antibodies to the transferrin receptor (TfR) was determined by their relative affinities at extracellular and endosomal pH. An antibody with reduced affinity at pH5.5 showed significant transcytosis, while pH-independent antibodies of comparable affinities at pH 7.4 remained associated with intracellular vesicular compartments and were finally targeted for degradation.

## Introduction

Despite decades of intensive research in the labs of academic institutions and the pharmaceutical industry, the blood-brain barrier has remained a significant hurdle for treatment of CNS diseases with growing unmet medical need [1]. Limiting brain access to small, predominantly hydrophobic molecules, this barrier is especially insurmountable to therapeutic proteins. Although antibodies are in clinical development for several CNS indications, brain exposure of these potential medicines is low (<0.1%) and may in many cases be insufficient for therapeutic efficacy. The most important physiological entry route for proteins into the brain is through receptor-mediated transcytosis (RMT), the exploitation of which has already been proposed for the transport of biologics into the brain [2]. However, the pathways and sorting mechanisms of transcytosis in blood-brain barrier endothelial cells are poorly understood, preventing the targeted generation of “brain shuttle” molecules. Another way to further our understanding on the molecular properties predisposing a protein, or more specifically an antibody, to efficient BBB passage, would be a robust *in vitro* transcytosis assay, enabling the screening of many antibodies and correlating their transcytosis capacity with other molecular properties like receptor specificity or affinity. Although many *in vitro* transcytosis assays have been described in the literature (for review see [3]), published data are often not in agreement with the calculated transcytosis capacity of brain endothelial cells, and may therefore rather represent paracellular flux than transcytosis. Additional requirements for a transcytosis assay to generate predictive data for clinical candidate development are the use of a human cellular system and robust, reproducible assay conditions with little inter-assay variability.

We sought to establish a reliable *in vitro* model system of transcytosis using the hCMEC/D3 immortalized human brain endothelial cell line [3], [4]. Although trans-endothelial electrical resistance (TEER) values indicate high paracellular flux of the confluent hCMEC/D3 cell layer, the cell line expresses endothelial specific and tight junction markers [4] and functional drug transporters [5] and has been successfully utilized as a surrogate for primary human brain endothelial cells in permeability studies [6], interaction of immune cells and pathogens at the BBB interface [7] or integrity of the BBB in neurodegenerative diseases [8], [9]. Indeed, the cell line has also been used to assess the transport of peptides across the endothelial cell monolayer albeit mostly with insufficient control of paracellular flux [10], [11]. We chose the transferrin (Tf): transferrin receptor (TfR) model system to validate our assay. The transferrin receptor (TfR) represents a prototypical receptor for RMT; its expression and function in capillary endothelial cells has been thoroughly investigated [12]–[14] and data from [15]–[17] strongly support the transcytosis of diferric transferrin across the endothelial cell and release into the brain parenchyma. Endosomal acidification after the internalization of the Tf-TfR complex leads to release of iron from the ligand, but apo-transferrin stays bound to the receptor until the complex is transported to the plasma membrane, where it is released due to poor binding affinity to the receptor at neutral pH.

We have successfully adapted the “pulse-chase” method described by Raub and Newton to eliminate experimental artifacts caused by the poor TEER and increased paracellular flux inherent to this cell line. We provide evidence for transcytosis of transferrin and show that co-culture with astrocytes does not influence uptake or transcytosis of the ligand. We have developed highly sensitive ELISAs for the detection of human, mouse and hamster IgG in picogram quantities, which would enable radiolabel-free evaluation of antibodies for transcytotic potential in a medium through-put format. We describe the transport potential of antibodies against TfR as well as other potential transcytosis receptors tested in the assay. While an antibody against the IGF1 receptor (IGR1R) was exclusively recycled to the apical compartment, certain anti-TfR antibodies were successfully transported across the endothelial cell layer, while others were targeted for degradation in intracellular compartments. Interestingly, we found a correlation between the transcytosis capacity of the anti-transferrin receptor antibodies and pH-dependence of the receptor-antibody interaction, identifying a potential new mechanism for the enhancement of transcytosis.

## Materials and Methods

### Antibodies

The cDNAs of heavy and light chains of antibody 128.1 (described in WO93/10819 and [18]) were cloned into pcDNA3.1-derived expression vectors and the antibody was expressed into the culture medium of HEK293F cells after transient transfection of both vectors according to a standard protocol (#K9000-01, Invitrogen, Frankfurt, Germany). The antibody was purified over a protein-A column and radiolabelled at Perkin Elmer (Rodgau, Germany). IGF-1R mAb R1507 is described in US7572897 and [19]. Antibody MEM-189 was obtained from Biozol (Eching, Germany), MEM-75 and 13E4 from Abcam (Cambridge, UK), M-A712 from BD Biosciences (Heidelberg, Germany) and LT-71 from Hytest (Turku, Finland).

### Cell Culture

hCMEC/D3 cells, a brain endothelial cell line immortalized by transduction with hTERT and SV40 large T [4] were obtained from Pierre-Olivier Couraud under license (INSERM, Paris, France). Medium and supplements for hCMEC/D3 and primary human astrocytes were obtained from Lonza (Verviers, Belgium). MDCK and rat C6 media and components were obtained from Invitrogen.

Primary human astrocytes (passages 2–4) were obtained from Cell Systems (Troisdorf, Germany) and cultured in ABM medium fully complemented with the AGM SingleQuots kit. Rat C6 Glioma cells from ATCC were cultured in DMEM-F12 containing 5% heat inactivated FBS, 10% horse serum and 2 mM L-Glutamine. MDCK cells from ATCC were cultured in MEM containing 10% fetal bovine serum, 2 mM L-glutamine, 1 mM sodium pyruvate, and 1500 mg/L sodium bicarbonate.

hCMEC/D3 cells (passages 26–29) were cultured to confluence on collagen (Sigma, Schnelldorf, Germany) coated coverslips (microscopy) in EBM2 medium containing 2.5% FBS, quarter of the supplied growth factors and fully complemented with supplied hydrocortisone, gentamycin and ascorbic acid.

For all transcytosis assays, high density pore (1×108 pores/cm^2^) PET membrane filter inserts (0.4 µm, 12 mm diameter; Millipore, Schwalbach, Germany) were used in 12-well cell culture plates (Corning, Amsterdam, Netherlands). Optimum media volumes were calculated to be 400 µl and 1600 µl for apical and basolateral chambers respectively. Apical chambers of Millicell hanging filter inserts were coated with Rat tail Collagen 1 (7.5 µg/cm^2^; BD Biosciences) followed by Fibronectin (5 µg/ml; Sigma) each incubation lasting for 1 hr at RT. hCMEC/D3 cells were grown to confluent monolayers (∼2×10^5^ cells/cm^2^) for 10-12 days in EMB2 medium. Empty filters coated with collagen/fibronectin were blocked in PBS containing 1% BSA o/n before the assay and then calibrated for at least 1 h in EBM2 before the assay. For generating endothelial-astrocyte co-cultures, the basolateral side of the filter inserts and TC plastic were coated with 10 µg/ml poly-d-lysine (Sigma) o/n at RT. The solution was aspirated, TC plastic dried for 2 h at RT and the coated surfaces were rinsed 3 × with PBS before astrocytes were seeded at 1×10^3^ in 200 µl on the bottom side of an inverted filter insert. Cells were allowed to adhere for 15 min at 37°C and the insert was then placed in a 12-well cell culture plate with the respective volumes of medium in apical and basolateral chambers. For culture of astrocytes in a culture plate, 1×10^3^ cells were plated in 1600 µl. After 48 hours the filters were processed for culturing hCMEC/D3 cells as described above. Experiments with the co-culture model were performed 10–12 days after hCMEC/D3 cells were seeded.

## Transcytosis assays

### 

#### Transferrin Transcytosis Assays

^125^I-Tfn was obtained from Perkin Elmer. The entire assay was performed in serum free EBM2 medium which was otherwise reconstituted as described. On day of the assay, cells were serum starved for 60 min to deplete Tfn. Filter inserts with or without cells were incubated apically with radiolabelled ligand for 1 h at 37°C following which the entire apical and basolateral volumes (referred to as values of stock post loading) were collected. Paracellular flux and stability of the radio-iodinated ligand were calculated from these values. The monolayers were washed at RT in serum free medium apically (400 µl) and basolaterally (1600 µl) 3 x, 3–5 min each. All the washes were collected to monitor efficiency of removal of the unbound ligand. Prewarmed medium was added to the apical chamber and the filter insert transferred to a fresh 12 well plate (blocked o/n with PBS containing 1% BSA) containing µl pre-warmed medium. At this point, filters with or without cells were lysed in 500 µl RIPA buffer in order to determine specific ligand uptake. The remaining filters were incubated at 37°C or at 4°C and samples collected at various time points to determine apical and/or basolateral release of ligand. Intact and degraded Tfn was assessed using TCA and AgNO_3_ precipitation as follows: Media and cell lysates were centrifuged in the presence of trichloroacetic acid and carrier protein (BSA) at 1100 × g for 10 min. Intact protein is associated with the pellet, degraded protein and free iodine remains in the supernatant, which is then centrifuged in the presence of AgNO3 at 1100 × g for 10 min to separate degraded protein (supernatant) from free iodine (AgI precipitate). For each time point, data was generated from two empty filter inserts and three inserts with cell cultures.

#### Antibody Transcytosis Assays

The entire assay was performed in complete EBM2 medium and protocol similar to that of the ligand based assays except for the detection of IgG in transcytosis assay supernatants by ELISA. The entire procedure was performed at RT and Biotek ELx405 was used to perform the wash steps. Briefly, a 384 well plate was coated with 30 µl/well of 1 µg/ml anti-human/mouse-IgG, Fcγ-specific (Jackson Immunoresearch, Newmarket, England) in PBS without Ca2+ and Mg2+ (Invitrogen) for 2 h followed by an hour's incubation in blocking buffer (Roche; PBS containing 1% BSA or 1% Crotein C for human and mouse IgG assays respectively). Serially diluted samples from the transcytosis assay and standard concentrations of the antibody used in the transcytosis assay were added to the plate and incubated for 2 h. After four washes, 30 µl/well of 50 ng/ml anti-human/mouse-F (ab) 2-Biotin in blocking buffer was added and incubated for a further 2 h. Following 6 washes, 30 µl/well of 50 ng/ml (huIgG assay) or 100 ng/ml (mIgG assay) Poly-HRP40-Streptavidin (Fitzgerald, Acton/MA, USA; in PBS containing 1% BSA and 0.05% Tween-20) was added and incubated for 30 min. After 4 washes, immune complexes were detected by addition of 30 µl/well of BM Chemiluminescence Substrate (Roche). The luminescence signal was measured using TECAN F200 and concentration calculated using the fitted standard curve. The sensitivity of the assay ranged from 10 pg/ml to 10 ng/ml.

### Immunofluorescence using Confocal Microscopy

To investigate the localization of the 128.1 antibody and the natural ligand holotransferrin, monolayers of hCMEC/D3 cells grown to confluence on collagen-coated cover slips were incubated with 5 µg/ml FITC tagged holotransferrin (Invitrogen) or 1 µg/ml 128.1, MEM-189 or the IGF-1R antibodies for 10 min following which the medium was removed and replaced with fresh medium. After 1 hour at 37°C, the monolayers were fixed in 4% PFA for 15 min at RT, permeabilized for 10 min (1× PBS with 0.1% Triton X-100) and incubated with an antibody to late endosomal/lysosomal marker CD63 (Abcam, Cambridge, UK) for 1 hour at RT. Cells were subjected to 1× PBS washes for 15 min and sequentially incubated where necessary with secondary antibodies; goat anti-human IgG-Alexa Flour 488 and/or chicken anti-mouse IgG-Alexa Fluor 595 (1∶200, Invitrogen) for 1 hour at RT. Cells were washed in 1× PBS for 30 min and cover slips mounted in UltraCruz fluorescent mounting medium (Santa Cruz, Heidelberg, Germany) and confocal fluorescent images were obtained using an Leica DM IRB/E microscope. Confocal images and z-stacks were acquired and analyzed by Leica Confocal Software (Leica, Wetzlar, Germany. All the confocal images show a single, representative, section of a Z-series taken through the entire cell.

### Binding and Competition ELISA

The entire procedure was performed at RT on a shaking platform and all wash steps (PBS containing 0.1% tween) were done on the Biotek ELx405. Briefly, a 96 well plate (Nunc, Langenselbold, Germany) was coated for 1 h with 50 ng of the extracellular subunit of recombinant TfR (R&D Systems, Wiesbaden, Germany). The plates were then blocked for 1 h in sample buffer (PBS containing 1% BSA) and washed 4 x. 50 µl of antibody dilutions made in sample buffer were transferred to the assay plate and incubated for 1.5 h following which the plates were washed 4 x. Peroxidase labeled anti mouse IgG (GE Healthcare, München, Germany) or anti human IgG (Jackson Immunoresearch) secondary antibodies used at recommended dilutions were added to the wells and incubated further for 30 min. Following 6 washes, 50 µl of TMB (Sigma) was added per well and incubated for 5–20 min. After addition of 50 µl of 1N HCl, antibody-receptor complexes were detected by measuring the OD of the plate at 450/620 nm. The procedure for the blocking and competition ELISA was similar except in the latter assay, dilutions of respective antibodies in sample buffer were added to the plate for 1 h in the pre-block conditions and assay plates washed 4 × after this incubation.

## Results

### “Pulse-chase” assay mode overcomes leakiness in hCMEC/D3 transcytosis assay

**Figure 1:**
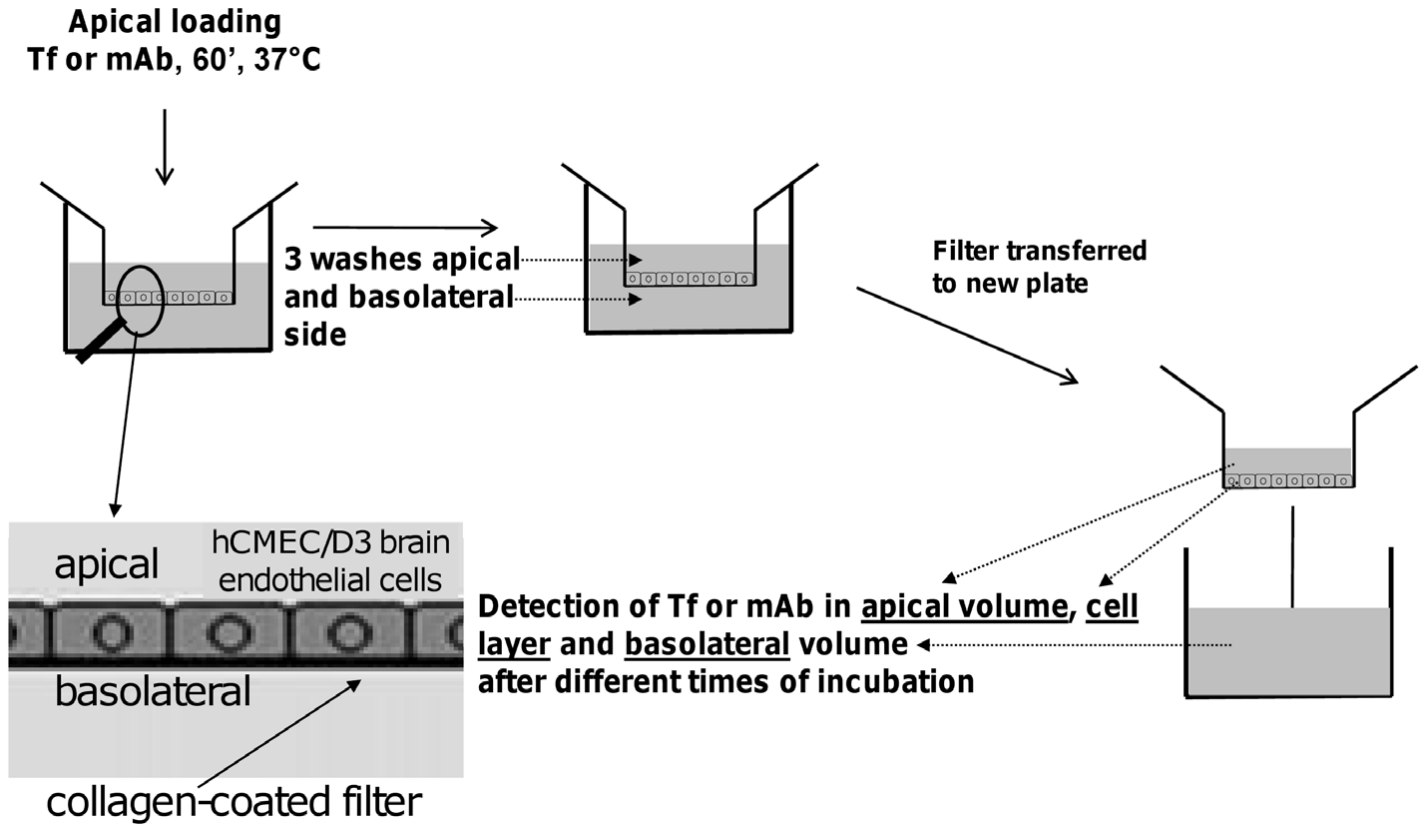
Description of the transcytosis assay. hCMEC/D3 cells grown to confluence on collagen and fibronectin coated membrane filter inserts (and serum starved for 1 h at 37°C before assay for experiments with ^125^I-transferrin) were incubated apically with the ligand or the different antibodies for 60 min at 37°C. After 1 hr, media from the apical and basolateral chambers were collected to assess paracellular flux following which the luminal and abluminal membranes of the monolayer were washed four times with medium at RT and the washes monitored to determine efficiency of removing unbound antibody. The filters containing cells were transferred to a fresh plate containing pre-warmed medium and cells were chased up to the desired time points at 37°C or on ice and at these different time points, the ligand or antibody associated with cells or in the media from the apical and basolateral chambers was analyzed in a gamma counter or assessed by IgG ELISA.

hCMEC/D3 cells were cultured to confluent monolayers on collagen/fibronectin-coated filter inserts, and upon reaching the TEER value of ≈40 ohm/cm^2^, were used to investigate the apical-to-basolateral transport of transferrin. On the basis of kinetic parameters obtained with iodinated transferrin in primary bovine brain endothelial cells (influx rate of 0.03 min^−1^, total efflux of 0.017 min^−1^ and a ratio of 1∶3 between basolateral and apical efflux) [16], we calculated an expected transport rate of 62 pg of the ligand per hour in our assay set-up. However, the paracellular flux of the hCMEC/D3 cellular monolayer to 70 kDa FITC dextran is 1.8 × 10^−5^ cm/min, which would equate to 6.8 ng/mL of the ligand in the basolateral compartment following an hour's incubation with 1 µg/mL ^125^I-Transferrin. This indicated that it would be impossible to measure transcytosis against the expected background of paracellular flow. Indeed when filter-grown hCMEC/D3 cells were apically incubated with 1 µg/mL ^125^I-Transferrin for 1 hour, close to 10,000 counts (∼ 30 ng) of the ligand were measured in the basolateral compartment. In filters without cells, this value was elevated by a factor of seven, still demonstrating restriction to protein passage by the cellular monolayer. Since the internalized ligand measured in cellular lysates was in the range of 5-7 ng in several experiments and unchanged by the concentration of the ligand used, it is likely that the events thus measured in the basolateral compartment are mostly contributed by paracellular flux and not by transcytosis. We confirmed this possibility by using ^125^I-Protein A as a negative control (data not shown). We did not detect radiolabel associated with the hCMEC/D3 cells, but the radiolabel measured in the basolateral compartment was similar to the assay with iodinated Tf under the same conditions. These results confirmed the necessity of modifying our assay to a “pulse-chase” format as suggested by Raub and Newton (Fig. 1).

**Figure 2:**
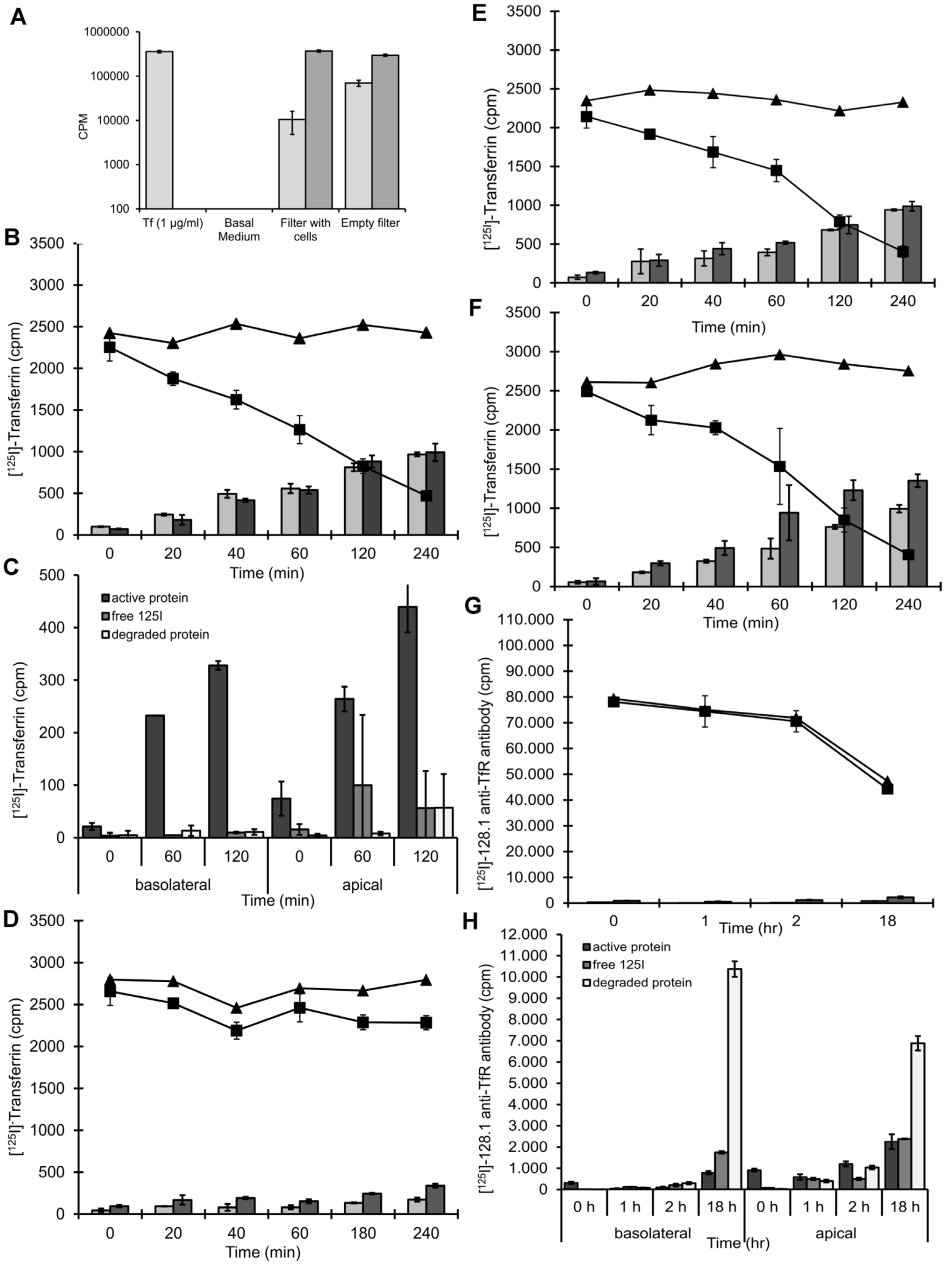
Apical to basolateral transport of ^125^I-Tfn and ^125^I-128.1 anti-TfR antibody in hCMEC/D3 cells. **A**: Empty filters (Empty Filters) treated as described in Materials and Methods or hCMEC/D3 cells grown to confluence on collagen and fibronectin coated membrane filter inserts (filters with cells) (serum starved for 1 h at 37°C before assay) were incubated apically with ^125^I-transferrin (Tf stock 1 µg/mL) for 60 min at 37°C. After 1 hr, radioactivity associated with media from the apical (*dark grey bars*) and basolateral chambers (*grey bars*), was analyzed in a γ counter. **B, C, and D**: hCMEC/D3 cells grown to confluence on collagen and fibronectin coated membrane filter inserts were serum starved for 1 h at 37°C and then incubated apically with 1 µg/mL ^125^I-transferrin (in serum free medium) for 60 min at 37°C. Cells were washed to remove unbound ligand and chased up to 4 hours at 37°C (**B**) or on ice (**D**). At the end of the chase, radioactivity associated with cells (▪) and media from the apical (*grey bars*) and basolateral chambers (*black bars*), was analyzed in a gamma counter at different time points. The total amount of the radiolabelled ligand (▴) derived from combined value of radiolabel in the lysate and in the media chambers remained constant during the assay. Each data point represents values obtained from three filter membrane inserts with cells and these results are representative of three separate experiments. (**C**), denotes active protein (*black bars*), degraded protein (*white bars*) and free iodine (*grey bars*) obtained from TCA precipitation of apical or basolateral media compartments from (**B**). **E and F**: hCMEC/D3 cells grown to confluence in a contact co-culture with primary human astrocytes (**E**) or in a non-contact culture condition with the rat C6 cell line (**F**) were incubated for 1 h at 37°C in serum free media to deplete intracellular transferrin. Cultures were incubated apically with 1 µg/mL ^125^I-transferrin (in serum free medium) for 60 min at 37°C. Cells were washed to remove unbound ligand and cultured at 37°C. Apical to basolateral transport of the ligand (*black bars*) or apically recycled (*grey bars*) internalised ligand was determined by analyzing the whole cell lysates (▪) and the media from the apical and basolateral chambers in a gamma counter at different time points. The total amount of the radiolabelled ligand (▴) derived from combined value of radiolabel in the lysate and in the media chambers was constant during the assay. Values are means of three filters ± SEM. **G and H**: hCMEC/D3 cells grown to confluence on collagen and fibronectin coated membrane filters were incubated apically with 6.5 µg/mL ^125^I-128.1 (**G**) in a radiolabel assay format for 60 min at 37°C. Luminal and abluminal membranes of the monolayer were washed four times with medium at RT and the washes monitored to determine efficiency of removing unbound antibody. Cells were chased up to 18 hrs at 37°C. At the end of the chase, radioactivity associated with cells (▪) and media from the apical (*grey bars*) and basolateral chambers (*black bars*), was analyzed in a gamma counter at different time points. The total amount of the antibody (▴) derived from combined value of radiolabel in the lysate and in the media chambers was calculated for the duration of the assay. Each data point represents values obtained from three filter membrane inserts with cells and these results are representative of more than three separate experiments. (**H**), denotes active protein (*black bars*), degraded protein (*white bars*) and free iodine (*grey bars*) obtained from TCA precipitation of apical or basolateral media compartments from (**G**).

Serum starved cells were incubated with iodinated transferrin for one hour at 37°C to allow for ligand internalization. After washing and transferring of the filter to a fresh plate, the amount of radioactivity in the apical and basolateral medium compartments as well as in the cell lysate was determined by gamma-counting at different incubation times. After four hours of incubation, approximately 40% of the radioactivity internalized by the cells was observed at the apical side (recycling), 40% at the basolateral side (transcytosis), and 20% remained inside the cells (Fig. 2B). As shown by trichloro-acetic acid (TCA) precipitation), >90% of the transported material was active protein, excluding the leakage of radioactivity after protein degradation (Fig. 2C). When transcytosis was carried out at 4°C, only minimal amounts of radioactivity were found in the basolateral and apical compartments, confirming the implication of an energy-dependent, active transport process. Although hCMEC/D3layer tightness has been shown not to respond to cues delivered by astrocytes, we tested if transferrin transport might be influenced by the presence of rat or human astrocytes in a contact or non-contact culture (Fig. 2E and F); however, this was not the case.

Owing to the high serum concentration, transferrin is a sub-optimal brain transporter and so we tested an antibody to the human transferrin receptor (128.1), described by Friden et al. [18] to access the brain of Cynomolgus monkeys following intravenous injection. In our transcytosis system, ^125^I-labeled 128.1, although readily taken up by the cells, even after 18 hours was not transported to either basolateral or apical side, but remained in cells (Fig. 2G). TCA precipitation confirmed that a large portion of the antibody was degraded inside cells (Fig. 2H).

### Transcytosis of antibodies against BBB receptors reveals details about sorting mechanism

**Figure 3:**
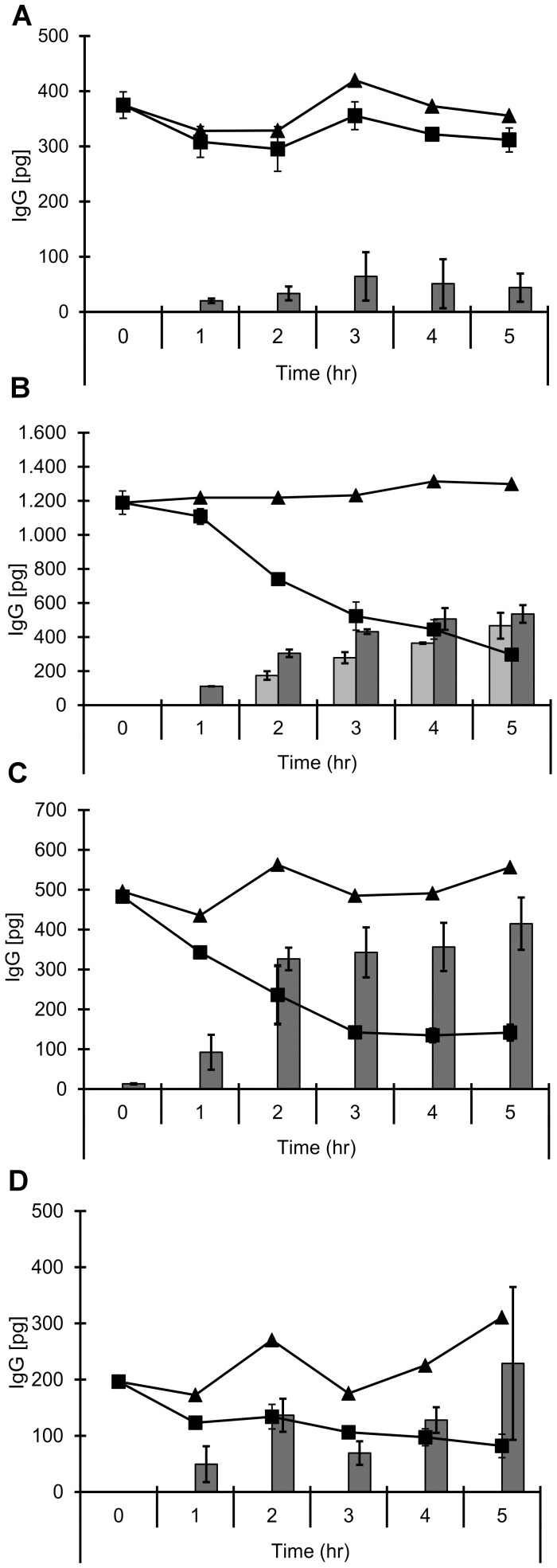
Uptake and fate of an antibody directed against different transcytosis receptors in hCMEC/D3 monolayers. **A–D** hCMEC/D3 cells grown to confluence on collagen and fibronectin coated membrane filters were incubated apically with 1 µg/mL of the following antibodies: the anti-human TfR antibody, 128.1 (**A**), the mouse anti–human TfR antibody, MEM-189 (**B**), an anti-human IGF-1R antibody (**C**) and the mouse anti-human insulin receptor (IR) antibody, 83–14 (**D**) for 60 min at 37°C. Luminal and abluminal membranes of the monolayer were washed four times with medium at RT and the washes monitored to determine efficiency of removing unbound antibody. Cells were chased up to 5 hrs at 37°C. At the end of the chase, antibody associated with cells (▪) and media from the apical (*grey bars*) and basolateral chambers (*black bars*), were analyzed by the sensitive IgG ELISA at different time points. The total amount of IgG (▴) derived from combined value of antibody present in the lysate and in the media chambers was calculated for the entire duration of the assay. Values are means of three filters ± SEM.

Next, we established highly sensitive IgG ELISAs in order to be able to detect picogram quantities of transcytosed IgG, obviating the need for radioactive labeling. Figure 3A shows that using this IgG ELISA, results for mAb 128.1 transcytosis were very similar to those obtained with radioactive antibody. We then went on to test other antibodies in our transcytosis system. A commercially available antibody against the transferrin receptor, mAb MEM-189, to our surprise showed a completely different behavior compared to mAb 128.1 (Fig. 3B): after five hours of incubation, about 40% of the antibody were found in each the basolateral and the apical compartment, while only 20% had remained inside cells, indicating that the antibody had been transcytosed and recycled to equal amounts, in a similar way as shown for the ligand, transferrin. By contrast, an antibody against the IGF1 receptor, although capable of leaving the endothelial cells, was exclusively recycled to the apical compartment (Fig. 3C), while an antibody against the insulin receptor showed only weak uptake and some recycling (Fig. 3D). In conclusion, our assay proved to be a valuable tool for the investigation of BBB sorting pathways for different internalizing receptors.

**Figure 4:**
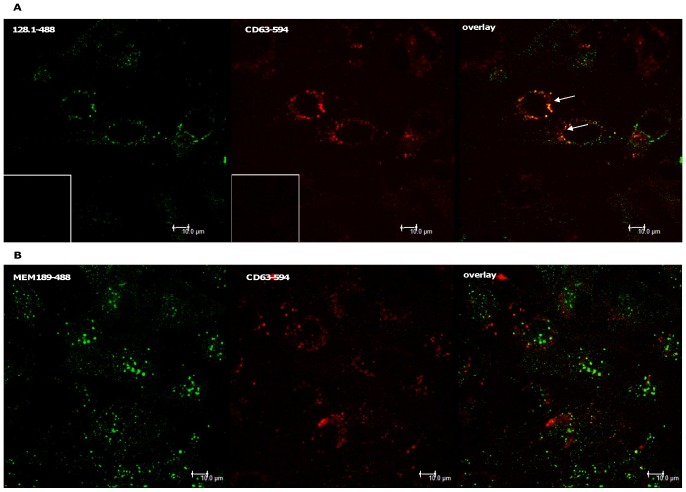
Degradation of 128.1 but not MEM-189 in hCMEC/D3 cells. hCMEC/D3 endothelial cells were pulsed with 1 µg/ml 128.1 (**A**) or MEM-189 (**B**) for 10 min at 37°CThe coverslip cultures were washed and cultured at 37°C for various time periods. Cultures were fixed in 4% PFA, permeabilised and immunostained with an antibody to the late endosomal/lysosomal marker CD63 and appropriate secondary antibodies as described in Materials and Methods and examined with a laser scanning confocal microscope. Insets: secondary antibodies only.

In order to have a thorough understanding of the intracellular fate of the two TfR antibodies mAb 128.1 and mAb MEM-189, hCMEC/D3 cells were pulsed with each of the antibodies for ten minutes, unbound antibody was removed and after one hour of incubation, cells were fixed and stained for immunofluorescence microscopy. Figure 4 shows the intracellular localization of the antibodies together with a staining of CD63, a marker for the late endosomal/lysosomal compartment. While mAb 128.1 shows a significant co-localization with CD63, MEM-189 was found in vesicular structures different from late endosomes. These observations are in line with the transcytosis assay data, arguing for intracellular degradation of mAb 128.1 and differential sorting of MEM-189 to recycling and transcytosis pathways.

**Figure 5:**
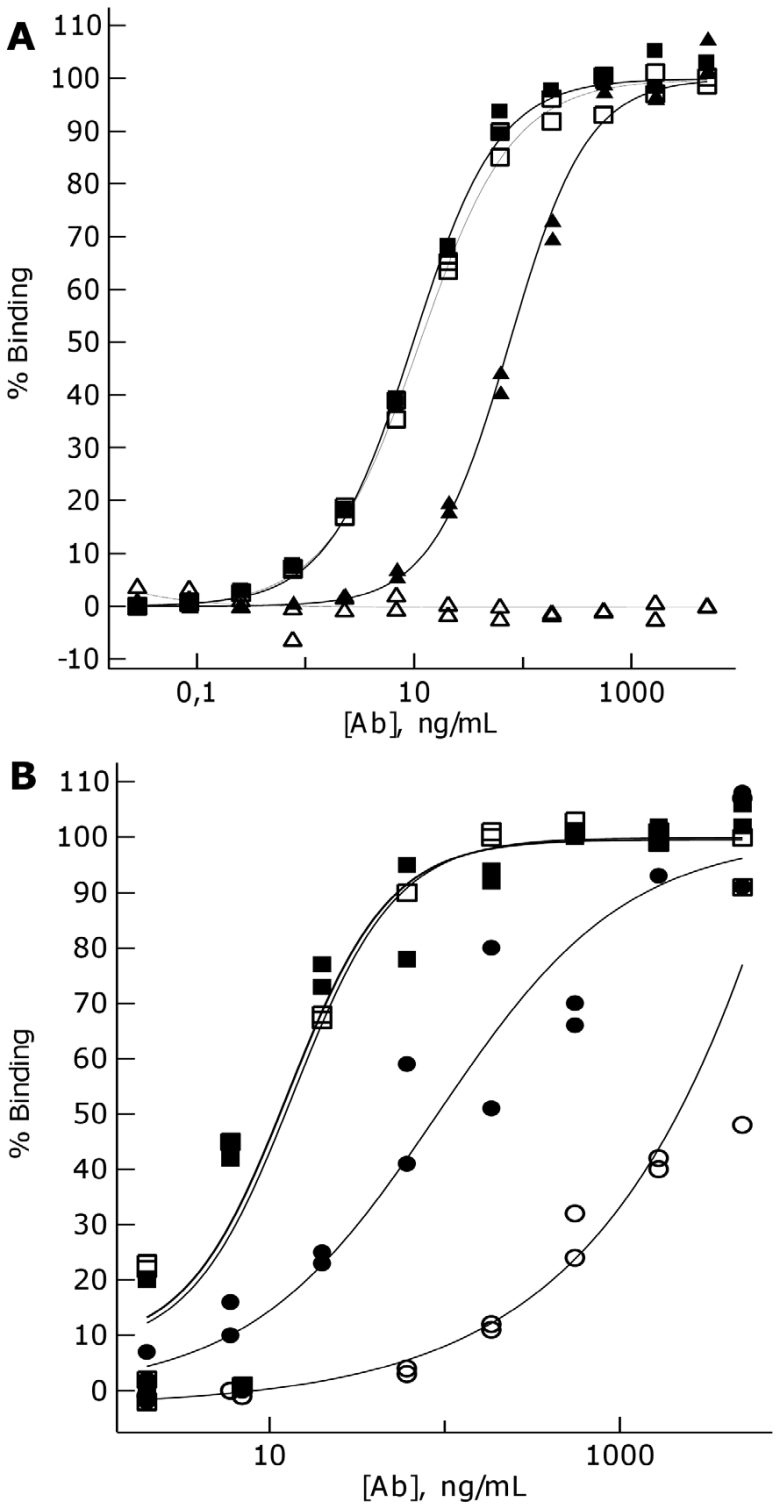
128.1 is a high affinity antibody that competes successfully with MEM-189 for binding to TfR in ELISA and in contrast to MEM-189 shows no pH dependence. **A**: Competition ELISA described in Materials and Methods showing binding of 128.1 to the extracellular subunit of TfR in the presence (▪) or absence (▴) of a pre-block by 5 µg/ml MEM-189. Similarly, binding of MEM-189 in the presence (□) or absence (▵) of 5 µg/mL 128.1. **B**, Binding ELISA described in Materials and Methods showing binding of the 128.1 anti-human transferrin receptor antibody (▪,□) and MEM-189 antibody (•,○) to the extracellular subunit of the human TfR at pH 7.4 (▪,•) or at pH 5.5 (□,○).

Next we asked the question, what property of the antibodies might be responsible for their intracellular fate. In order to compare binding epitopes on the human TfR extracellular domain (ECD), we performed a competition ELISA on immobilized TfR-ECD (Fig. 5A). mAb 128.1 was not inhibited from binding to TfR-ECD, but pre-incubation of TfR-ECD with 128.1 completely abolished binding of MEM-189. The significantly higher affinity of 128.1 as compared to MEM-189 could explain why blocking is not efficient in both ways. However it was clear that the epitopes of the antibodies are overlapping and unlikely to reside in completely different regions of the receptor, which could exclude the antibody epitope as a determinant of intracellular sorting. Affinity itself, as shown by Yu et al., may be one of the criteria influencing transcytosis, but we later identified antibodies in the same affinity range as MEM-189, not capable of transcytosis (see below). We speculated that pH change during acidification of endosomes may play a role in TfR antibody sorting, and indeed we could show that MEM-189 showed a strongly diminished affinity for the TfR at pH 5.5 (late endosomal pH), as compared to pH 7.4 (Fig. 5B). mAb 128.1, by contrast, was pH-independent.

**Figure 6:**
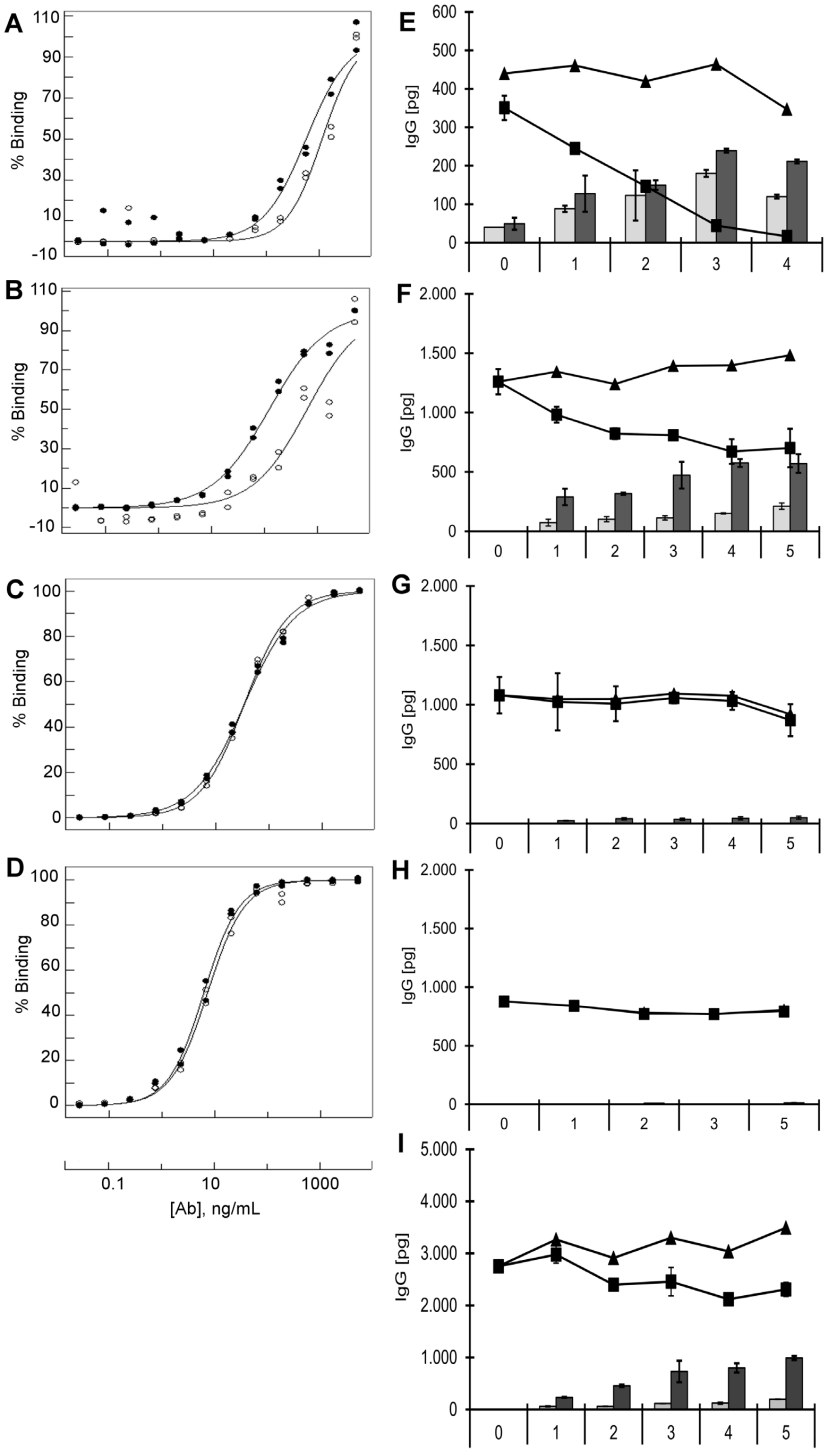
pH dependence and uptake, fate of different antibodies directed against the human TfR in hCMEC/D3 monolayers. **A–D**: Binding ELISA described in Materials and Methods showing binding of mouse anti-human transferrin receptor antibodies LT-71 (**A**), MEM-75 (**B**), M-A712 (**C**) and 13E4 (**D**) to the extracellular subunit of TfR at pH 7.4 (•) or at pH 5.5 (○). **E**–**I**: hCMEC/D3 cells grown to confluence on collagen and fibronectin coated membrane filters were incubated apically with 1 µg/mL of the following mouse anti-human TfR antibodies: LT-71 (**E**), MEM-75 (**F**), M-A712 (**G**), 13E4 (**H**) for 60 min at 37°C. Luminal and abluminal membranes of the monolayer were washed four times with medium at RT and the washes monitored to determine efficiency of removing unbound antibody. Cells were chased up to 5 hrs at 37°C. At the end of the chase, antibody associated with cells (▪) and media from the apical (*white bars*) and basolateral chambers (*grey bars*), were analyzed by the sensitive IgG ELISA at different time points. The total amount of IgG (▴) derived from combined value of antibody present in the lysate and in the media chambers was calculated for the entire duration of the assay. Values are means of three filters ± SEM. (**I**) hCMEC/D3 cells were incubated apically with 1 µg/mL mouse anti-human TfR MEM-189 antibody as in (**E**–**H**) and cells were chased up to 5 hrs at 37°C in the presence of 50 nM Bafilomycin. At the end of the chase, antibody associated with cells (▪) and media from the apical (*grey bars*) and basolateral chambers (*white bars*), were analyzed by the sensitive IgG ELISA at different time points. The total amount of IgG (▴) derived from combined value of antibody present in the lysate and in the media chambers was calculated for the entire duration of the assay. Values are means of three filters ± SEM.

Testing other commercially available antibodies for pH-dependent binding to the TfR, we observed that antibodies LT-71 and MEM-75 displayed weaker binding to the receptor at endosomal pH, while antibodies M-A712 and 13E4 bound in a pH-independent fashion (Fig. 6, A–D). Strikingly, the latter two antibodies were irreversibly trapped inside the hCMEC/D3 cells when investigated in our transcytosis assay (Fig. 6, G and H), while LT-71 and MEM-75 showed different degrees of transcytosis and recycling (Fig. 6, E and F). Although these data also show a correlation between affinity and transcytosis, the comparable affinities of antibodies M-A712 and MEM-189, combined to their strikingly different transcytosis behavior, indicate that there may be additional mechanisms governing the intracellular fate of transcytosing antibodies. Finally, we wanted to investigate if transcytosis of pH-dependent TfR antibodies could be blocked by bafilomycin, an inhibitor of endosomal acidification. Figure 6I shows that pre-incubation of hCMEC/D3 cells with bafilomycin strongly reduced basolateral passage of antibody MEM-189, while apical recycling was unaffected. This result confirmed our hypothesis that endosomal acidification is an essential mechanistic step in facilitating the transcytosis of TfR antibodies with reduced affinity at low pH.

## Discussion

There is a wealth of literature describing the phenomenon of transcytosis *in vivo* and *in vitro* in addition to the classical pathway of receptor-mediated endocytosis and recycling. However, many publications describing *in vitro* models of protein transcytosis through the blood-brain barrier neglect the magnitude of paracellular flux as opposed to the small amount of transcytosed material [20]–[22]. In fact, our results show (Fig. 2A) accumulation of 30 ng of the transferrin ligand or Protein A in the basolateral compartment following incubation with 1 µg/mL of the radiolabel. In order to cope with this limitation, which is especially apparent in the relatively leaky monolayers of hCMEC/D3 cells [4], we have applied a pulse-chase assay set-up, initially described by Raub and Newton for primary bovine brain endothelial cells [16]. In order to detect the low amounts of transcytosed antibody and at the same time avoid using radiolabeled material, the development of highly sensitive IgG ELISAs has been instrumental for assessing the transcytosis potential of antibodies of different species. Furthermore, the ELISA protocol can be easily adapted for automation which makes it highly attractive in terms of assay throughput.

Although several studies addressed the transcytosis of antibody-targeted nanoparticles and immunoliposomes across hCMEC/D3 [10], [23], this is the first study to investigate the transcytosis and the fate of free antibodies targeting receptors capable of mediating transcytosis in immortalized human brain endothelium. Our results support the following conclusions:

Apical to basolateral transcytosis of intact transferrin occurs in hCMEC/D3 monolayer cultures. Ligand is also equally recycled to the luminal membrane. Both events are temperature sensitive but not modulated by astrocyte co culture (Fig. 2)

An antibody to the IGF1R is exclusively recycled, while antibodies against the TfR are either degraded in lysosmes or recycled/transcytosed (Figs. 3 and 4)

Reduced affinity of antibodies to the transferrin receptor at endosomal pH may enhance antibody transcytosis (Figs. 5 and 6).

We provide strong evidence for the transcytosis of intact transferrin in hCMEC/D3 cells (Fig. 2C–D). Between several experiments, we recorded similar values (2200–2500 cpm; 5–7 ng/∼20 µg cell protein) of uptake followed invariably by equivalent rates of transcytosis and recycling of the internalized ligand. High values of transcytosed/recycled ligand (∼5 ng) from our assay can be explained by the level of membrane-localized TfR on hCMEC/D3 cells compared to primary human brain endothelial cells. At similar culture confluency, the immortalized cells consistently show higher levels of membrane-resident TfR than primary human endothelial cells (data not shown). The work of Raub and Newton (1991; [16]) and Descamps et al. (1996; [15]) have described the paucity of membrane-localized TfR in confluent cultures of primary endothelial cells and hence the published values of transcytosis from primary cell cultures are generally low. The ratio of transferrin transcytosis to recycling was described as 1∶3 in primary bovine brain endothelial cells [16]; by contrast, we observe similar rates for both processes. It cannot be excluded that this difference, in line with the higher TfR expression level, could also be due to slightly distorted sorting of the transferrin receptor in the immortalized cell line; alternatively, we cannot rule out the possibility that some material unspecifically bound to the cells and is only released after prolongued incubation.

Following our validation of the assay protocol with the ligand, we proceeded to test antibodies against putative transcytosis receptors for transport. Targeting of receptors, particularly the insulin and transferrin receptors by chimeric peptides and antibodies has been suggested to be an effective way of delivering drugs to the brain in several animal models [24]–[28]. The OX-26 murine monoclonal antibody to the rat TfR [29] and the 83–14 murine monoclonal antibody to the human insulin receptor [27] are the best known examples of antibodies with published BBB permeability properties.

We tested an antibody to the IGF-1R, described for its capacity to engage in transcytotic activity [28], [30]. Our results indicate slight intracellular degradation and significant recycling of the antibody to the apical surface (Fig. 3C), but no transcytosis. Investigation of IGF-1R mediated transcytosis has implicated facilitation of the process by the association with LRP1 in the rat brain [30], [31]. We could not detect membrane-resident LRP1 in hCMEC/D3 cells (data not shown). Reports showing Lrp1 expression in hCMEC/D3 were obtained using methods which detect both extracellular and intracellular protein [32]. Absence of membrane LRP1 could offer an explanation for the lack of transcytosis of an IGF-1R mAb in our model system.

We generated the 128.1 anti-TfR antibody described in Friden et al. (1996; [18]), because it had been suggested to access the brain in Cynomolgous monkeys after intravenous application. In hCMEC/D3 cells, the antibody initially appeared to follow the classical uptake and internalization through clathrin-coated vesicles and subsequent localization in early endosomes. It is well established in numerous cell types that the TfR-ligand complex is endocytosed and rapidly recycled [33], [34]. In fact, the receptor-ligand complex is commonly used as a marker of early and recycling endosomes. However, the 128.1 antibody localized to a CD63-positive late endosomal/lysosomal compartment one hour after internalization, where it presumably is degraded. In contrast to 128.1, the mouse monoclonal antibody MEM-189 is processed like the ligand (Fig. 3B). It is endocytosed at a slightly slower rate than transferrin and the 128.1 antibody in hCMEC/D3 cells, which is likely attributable to the lower-affinity binding profile of the antibody (Fig. 5A). However, once internalized, the antibody is processed through the endocytic pathway with transcytosis and recycling kinetics comparable to that of transferrin (Fig. 2B and Fig. 3B). The 128.1 and MEM-189 antibodies target overlapping epitopes, as shown by a competition ELISA (Fig. 5A), which lowers the probability that the MEM-189 local epitope on TfR is solely responsible for its transcytosis potential, although it cannot be excluded. Even antibodies with closely overlapping epitopes can demonstrate fundamental functional differences, as illustrated for the CD20 antibodies GA101 and rituximab [35].

Intracellular degradation of TfR antibodies after endocytosis has been described by others [36], and the 128.1 antibody follows those examples in terms of lysosomal localization and degradation (Fig. 4A). Furthermore, incubation of murine lymphoma cells with full IgGs against the TfR has been shown to even downregulate surface expression of the TfR [36]. In contrast, Yu et al. (2011; [37]) have demonstrated that in vivo, antibodies against the transferrin receptor are transported into the brain to an extent inversely correlated to their binding affinity. We compared the bivalent affinities of the different TfR antibodies by direct ELISA and observed a similar correlation with regard to transcytosis potential (Figs. 3 and 6). A low-affinity antibody, LT-71 (EC50 of approx. 660 ng/mL) was capable of shuttling through hCMEC/D3 monolayer, albeit after low uptake (Fig. 6A), while the high-affinity antibodies 128.1 and 13E4 (EC50 of 10 ng/mL) remain inside the cells. However, two antibodies with intermediate binding affinities (MEM-189: 85 ng/mL and M-A712: 35 ng/mL) demonstrated significantly different sorting behavior: while MEM-189 was efficiently transcytosed and recycled (Fig. 3B), M-A712 was incapable of leaving the endothelial cells (Fig. 6G). It is unlikely that the small difference in binding affinity should be responsible for the striking difference in transcytosis potential.

The transferrin:transferrin receptor complex undergoes dramatic conformational changes upon endosomal acidification, which are partly driven by iron release from the ligand, but which are accompanied by significant changes in the TfR conformation [38]–[40]. These conformational changes might be responsible for the observed pH-dependence of several TfR antibodies. In addition, histidine residues in antibody CDR could contribute to pH-dependent target binding. The mechanism of transferrin transcytosis, especially which sorting events determine routing for basolateral transcytosis or apical recycling, have not yet been investigated. In polarized cells, TfR is known to be recycled via recruitment of the adaptor protein AP1B in the recycling endosome [41]. Apo-Tf stays bound to the receptor and follows its path back to the cell surface. A key difference between antibodies and the ligand interacting with the transferrin receptor is the bivalent nature of the TfR:antibody interaction. High-affinity, bivalent TfR antibodies are invariably sorted to lysosomes, possibly by interfering with TfR sorting via irreversible receptor crosslinking [36]. Although the exact mechanism of this sorting event is unknown, it seems plausible that antibodies with reduced affinity at endosomal pH might relieve receptor cross-linking due to lower complex stability, allowing the receptor to pursue its physiological sorting pathway. It needs to be stressed that transcytosis supported by pH-dependent receptor binding has so far only been demonstrated *in vitro* using the hCMEC/D3 model system. For a more general applicability, other sytems like primary brain endothelial cells and, more importantly, *in vivo* experiments, need to be performed.In summary, we have developed a human BBB transcytosis assay enabling us to quickly screen antibodies for putative brain shuttle receptors for their transcytosis potential. Furthermore, our data suggest a mechanism in addition to reduction of binding affinity, which might facilitate antibody transcytosis over the BBB, namely pH-dependent binding to a transcytosis receptor.
